# Evaluation of Green-Synthesized Cuprospinel Nanoparticles as a Nanosensor for Detection of Low-Concentration Cd(II) Ion in the Aqueous Solutions by the Quartz Crystal Microbalance Method

**DOI:** 10.3390/ma15186240

**Published:** 2022-09-08

**Authors:** Noha Al-Qasmi, Wafa Al-Gethami, Dalal Alhashmialameer, Sameh H. Ismail, Ahmed H. Sadek

**Affiliations:** 1 Chemistry Department, Faculty of Science, Taif University, Al-Hawiah, Taif City P.O. Box 11099, Saudi Arabia; 2 Faculty of Nanotechnology for Postgraduate Studies, Sheikh Zayed Campus, Cairo University, 6th October City, Giza 12588, Egypt; 3 Zewail City of Science, Technology and Innovation, 6th October City, Giza 12578, Egypt

**Keywords:** Cd(II), QCM, green synthesis, cuprospinel nanoparticles, sensor

## Abstract

Cd(II) heavy metal is an extremely dangerous hazardous material for both humans and the environment. Its high toxicity is the reason behind the examination of new techniques for detecting very small concentrations of Cd(II). Recently, Quartz Crystal Microbalance (QCM) has been one of the techniques that have been widely used to detect trace heavy metal ions in solutions. It is a simple, inexpensive, portable, and sensitive gravimetric sensor due to its quality sensitivity lowest to nanograms. In this work, Cuprospinel nanoparticles were synthesized through the green synthesis approach using *Psidium guajava* L. leaf extract as a reducing agent, which is the first scientific description to report the preparation of these nanoparticles by this method. Subsequently, the synthesized nanoparticles were subjected to the characterization of their crystallinity, structure, and morphology by the XRD, N_2_ adsorption–desorption, zeta potential, DLS, AFM, SEM, and TEM analyzers. The prepared Cuprospinel nanoparticles were evaluated as a nanosensor for the detection of the very low concentration of Cd(II) ions in aqueous solutions using the QCM technique. The results of the characterization proved that the Cuprospinel nanoparticles have formed in the nanoscale with sub-spherical shapes and particles size ranging from 20 to 80 nm. The BET surface area and pore size analysis revealed that the synthesized Cuprospinel nanoparticles possess a surface area of 47.3 m^2^/g, an average pore size of 1.5 nm, and a micropore volume of 0.064 cc/g. The QCM results demonstrated the success of the Cuprospinel nanoparticles sensor in detecting the tiny amounts of Cd(II) ions in the aqueous solutions with concentrations reaching about 3.6 ng/L.

## 1. Introduction

Nowadays, the use of heterogeneous catalysts (especially nano-catalysts) appears to be necessary for various branches of organic synthesis, whether from an economical or environmental and green chemistry perspective. This accounts for their notable characteristics, particularly recovery and reusability features [1,2,3]. Magnetic nanoparticles have recently attracted great interest due to their unique superparamagnetic characteristics and high surface area-to-volume ratio. One of the most crucial magnetic nanomaterials is spinel structure materials, which are frequently ferrites. Where the spinel ferrite nanoparticles provide several benefits for the treatment of wastewater [4,5].

Ferrite-based nanomaterials, which are typically characterized as iron oxides that contain one or more metals in their structure and exhibit ferrimagnetism features have been the subject of great research interest in the past [6]. These ferrites are divided into many groups according to their magnetic characteristics and crystal structure. Hexaferrite (hexagonal crystal structure, e.g., BaFe_12_O_19_ and SrFe_12_O_19_), orthoferrite (MFeO_3_), garnet (M_3_Fe_5_O_12_), where M stands for rare earth cations, and spinel ferrites are the most well-known kinds of ferrites [7]. The standard chemical formula for spinel ferrites is AB_2_O_4_, where A and B are metal cations that are located at tetrahedral and octahedral sites, respectively. In fact, ferric (Fe^3+^) must be included in the chemical formula for the compound to be referred to as spinel ferrite [8]. The compounds with a formula of MFe_2_O_4_ (where M can be any metal with an oxidation state of +2, such as Co^2+^, Cu^2+^, Fe^2+^, Mn^2+^, Ni^2+^, and Zn^2+^) are typical examples of spinel ferrites [9]. Spinel ferrites are frequently used in high-density storage media, ferromagnetic fluids, catalysts, magnetic drug delivery systems, magnetic separation, magnetic resonance tomography, gas sensors, and other applications [5,10]. One of the most significant magnetic materials is Cuprospinel or copper ferrite (CuFe_2_O_4_), which has a regular spinel structure with tetrahedral A- occupied by Cu^2+^ ions and Fe^3+^ ions occupied by B- of nanoparticles [11]. Contrarily, Cuprospinel nanoparticles have been widely used in several scientific sectors due to their exceptional magnetic and conducting properties, good chemical and thermal durability, and advantageous catalytic efficiency [12,13,14]. These ferrites have previously been prepared using a variety of wet synthesis techniques, including co-precipitation, thermal decomposition, sonochemistry, and solvothermal [15]. Most of these techniques guarantee successful outcomes in terms of crystalline particles with small sizes and the morphological control of the final product [16].

Numerous scientific studies have revealed non-toxic and affordable techniques for biologically synthesizing nanoparticles that can be utilized by multicellular and unicellular organisms such as yeasts, fungi, bacteria, actinomycetes, seaweeds, and plant extracts [17]. These synthesis techniques have produced functional biomolecules that can act as reducing and stabilizing agents for nanoparticles produced throughout the biosynthesis process. Additionally, it offers useful insights into biocompatibility while lowering harmful consumption, making them applicable to both biomedical and environmental applications [18]. Various approaches have recently been reported for the biosynthesis of nanomaterials via the green chemistry method using plant aqueous extracts [19]. Among these plants, *Psidium guajava* L. has been used to treat some diseases such as diabetes, diarrhea, hypertension, gastroenteritis, skin care products, and wound healing. Secondary metabolites like polyphenols and phenolic substances like flavonoids are abundant in guava leaf extract, making them act as efficient reducing agents [20].

Water pollution by heavy metal ions and textile dyes has become the worst, endangering both human health and ecosystems [21,22,23]. Water contamination by hazardous metal ions such as Cd(II), Cr(VI), Cu(II), Pb(II), and Zn(II), as well as pollution by a microorganism are contemporary problems, which results in serious risks in the water and develops into significant general medical concerns [24,25,26]. Because of its prolonged biological half-life, Cd(II) is one of the heavy metals that are most dangerous to both humans and the environment [27,28]. There are several different ways that cadmium infiltrated the environment, including through metal plating, batteries (nickel–cadmium batteries), pigments, alloys, and phosphate fertilizers [29,30]. When cadmium enters the body through inhalation, ingestion, or skin absorption, it severely damages human organs [31]. Humans are affected by cadmium in both the short and long term [32]. The short-term effects of exposure to cadmium include nausea, vomiting, diarrhea, dry mouth, convulsions, muscle cramps, impaired sensations, liver damage, stupor, renal abnormalities, and effects on the lungs, cardiovascular system, liver, and sensory system [33,34]. Cadmium has a long-lasting influence on various human organs, including the pancreas, liver, intestines, placenta, and lungs [35,36]. The WHO suggested a 3 µg/L cadmium ion as a maximum permissible limit in all sources of drinking water [37]. As a result, it is crucial to remove cadmium from wastewater, making a thorough research on this subject necessary. This focused the scientists’ efforts on the use of the most effective ways to remove these metals. This has depended on the interest in the effectiveness and performance of the processes in which the different materials are used to remove these heavy metals. Thus, various methods have been developed for the treatment of heavy metal-contaminated wastewater [38,39]. These methods include precipitation, buoyancy, oxidation, electrochemical oxidation, adsorption, reverse osmosis, dissipation, film filtering, biosorption techniques, membrane filtration, chemical precipitation, electrodialysis, and sensing [40,41,42,43,44,45,46,47,48,49,50,51,52,53].

Quartz crystal microbalance (QCM) is an ultra-sensitive mass balance device that depends on the piezoelectric properties of quartz where any mechanical deformation on its surface gives appropriate voltage due to oscillating at a defined frequency. The frequency of oscillation was affected by a tiny change in mass on the electrode surface. The frequency change was monitored in real-time to illustrate molecular interactions or reactions that have taken place at the electrode surface [54]. Therefore, the QCM is a reliable, simple, fast, ultra-sensitive, and specific QCM-based immunosensor, which is able to detect a small limit of molecules or ions compared to the traditional methods [55].

It may be indicated that the use of *Psidium guajava* L. leaf extract as a reducing agent for the synthesis of Cuprospinel nanoparticles has not been previously reported. Aqueous leaf extract was used in this work for the green synthesis of Cuprospinel nanoparticles. Additionally, this study is the first report on the use of these nanomaterials as nanosensors to monitor low-concentration cadmium ions in aqueous solutions.

Thus, this study aims to synthesize Cuprospinel nanoparticles by a green approach using the extract of *Psidium guajava* L. leaf. The prepared Cuprospinel nanoparticles were characterized for their size, morphology, and structure composition using different techniques for the instance XRD, N_2_ adsorption–desorption, zeta potential, DLS, AFM, SEM, and TEM. Then, the synthesized Cuprospinel nanoparticles were evaluated as a highly efficient and sensitive nanosensor for detecting the very low concentrations of Cd(II) ions in the aqueous solutions using QCM technology.

## 2. Materials and Methods

### 2.1. Materials

FeCl_3_.6H_2_O, CuCl_2_.6H_2_O, Cd(NO_3_)_2_.4H_2_O, and NaOH were purchased from Sigma-Aldrich and used without any purification. Double-distilled water was used as a solvent to prepare all solutions in this study.

### 2.2. Preparation of the Psidium Guajava L. Leaf Extract

Guava leaves (*Psidium guajava* L.) were purchased from a local market and were initially cleaned with tap water. They were then cleaned with double-distilled water to eliminate impurities and dust. After that, they were left to dry naturally for five days. In total, 6 g of the dried leaf was combined with 100 mL of double-distilled water to create the leaf extract, which was then boiled at 60 °C for 30 min. Thereafter, guava leaf extract was filtered and saved for future investigations [56].

### 2.3. Green Synthesis of Cuprospinel (CuFe_2_O_4_) Nanoparticles

Spinel CuFe_2_O_4_ NPs were synthesized using the co-precipitation method according to [57] with minor modifications. In total, 5 grams of FeCl_3_.6H_2_O and 3 grams of CuCl_2_.6H_2_O were dissolved in double-distilled water and heated on a hot plate for approximately 15 min at 50 °C. Then, with vigorous stirring, 10 mL of the guava leaf extract was added to the chloride solution. The pH was increased to 10 by the dropwise addition of a 0.5 M NaOH solution into the mixture. At 60 °C, the resulting mixture was stirred for two hours. The resultant NPs were then rinsed in double-distilled water and then calcined at 600 °C for two hours. Moreover, water extraction also indicates the presence of primary and secondary metabolites, the majority of which are polar in nature. Polar proteins and mono- and oligo-saccharides are examples of primary metabolites. However, as before mentioned, the polar secondary metabolites like glycosides and polyphenolics like flavonoids and tannins may be responsible for the reduction of metal ions to nanoparticles.

### 2.4. Characterization

Characterization was classified into three categories, namely, phase composition, morphology, and structuring. Our strategy for the characterization of synthesized Cuprospinel nanoparticles aims to determine the morphology, composition, surface area, pore size, charge, and particle size distribution of these nanoparticles using several analysis techniques. The morphological analysis was employed to identify the shape and topography of synthesized Cuprospinel nanoparticles. Therefore, some advanced high-resolution microscopes are used for this purpose such as Atomic Force Microscopy (AFM), Scanning Electron Microscopy (SEM), and Transmission Electron Microscopy (TEM). An AFM instrument (5600LS, Agilent technology company, Santa Clara, CA, USA) was used to study the topographic nature of the prepared Cuprospinel nanoparticles. The sample should be prepared before analysis by the AFM. Firstly, the sample was subjected to ultrasound waves for 2 h, using an ultrasonic probe sonicator (UP400S, Hielscher, Oderstraße, Teltow, Germany) under a condition of 59 kHz, an amplitude of 83%, and a cycle of 0.79 for 20 min. Finally, a thin film of the sample was created under a vacuum using a spin coater instrument (Laurell-650Sz) at 600 rpm. Moreover, Gwyddion software was used to analyze the AFM results. The AFM images and data profile were obtained at 1.5 µm × 1 µm using tapping mode imaging (Al tap, 0.5 In/S speed, I. gain 0.5 and P. gain 5). An SEM instrument (JEOL, JSM-6701F Plus, Peabody, MA, USA) was used to supply the surface morphology of the prepared Cuprospinel nanoparticles under an acceleration voltage of 18 kV and magnification of 80,000 and 160,000×. The size and shape of the Cuprospinel nanoparticles were provided by TEM (JEOL, JEM-2100 high-resolution, Peabody, MA, USA). Prior to TEM analysis, the Cuprospinel nanoparticles were added to double-distilled water and were sonicated for 20 min using an ultrasonic probe sonicator with 55 kHz, an amplitude of 55%, and a cycle of 0.55 for 10 min. Then, 5 to 10 microns of the dispersed mixture were dropped onto a carbon-coated copper grid. The composition part contained some techniques that confirmed the formation of Cuprospinel nanoparticles without any impurities descended from the synthesis method. Hence, X-ray diffraction (EQUINOX 1000, Thermo Scientific Co., Lafayette, CO, USA) was used to affirm the formation of Cuprospinel without any secondary product. The X-ray source was Cu Kα radiation with a current of 35 mA and an applied voltage of 39 kV. The 2θ angles ranged from 20 to 90° with a scan speed of 0.3°/min. The structural analyses were performed to provide information about the specific surface area (BET method), pore size properties (DA method), charge (zeta potential), and size distribution (DLS) for the prepared Cuprospinel nanoparticles. Thence, for these purposes, a N_2_ adsorption–desorption analyzer (Nova Touch 4L, Quanta Chrome, Boynton Beach, FL, USA) was used to determine the surface area and pore size of the Cuprospinel nanoparticles according to the BET and DA methods, respectively. Initially, Cuprospinel nanoparticles were degassed at 65 °C for 1 h. After reaching adsorption equilibrium, the adsorbed amount of gas was determined using the applied pressure and applying the universal gas law. Nitrogen gas has a cross-section area of 16.2 Å^2^/molecule, a bath temperature of 77.35 K, magnetic susceptibility of 2 × 1e^−29^ (mL/mol), a molecular weight of 28.0134 g, a critical temperature of 126.2 K, and supercritical ads, a critical pressure of 33.5 atm. The particle size (DLS method) and zeta potential of the synthesized Cuprospinel nanoparticles were measured and determined by the zeta seizer instrument (NanoSight NS500, Malvern Panalytical, Malvern, WR, UK).

### 2.5. Quartz Crystal Micro-Balance (QCM) Technique

**Figure 1:**
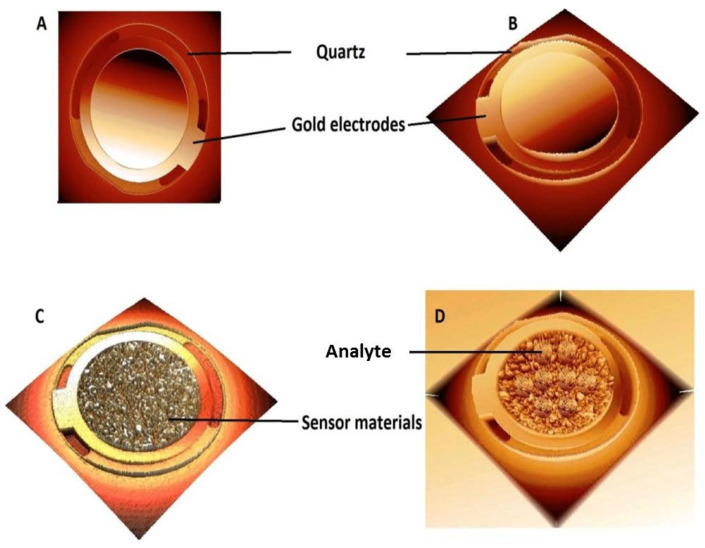
Illustrates (**A**) empty QCM detector (top view), (**B**) empty QCM detector (3D view), (**C**) QCM detector after the deposit of sensor material (Cuprospinel nanoparticles), and (**D**) QCM detector after attaching the analyte (Cd(II) ions).

A quartz crystal microbalance methodology was used to evaluate the efficiency of Cuprospinel nanoparticles as nanosensor material for detecting the Cd(II) ions in the aqueous solutions at low concentrations. Accordingly, for this procedure, a QCM (Q-senses, Biolin Scientific, Linthicum Heights, MD, USA) with gold electrodes was employed. The QCM was integrated with a flow meter, which was utilized to deliver the nanosensor material (100 µg of Cuprospinel nanoparticles was dispersed in 100 mL of double-distilled water) above the QCM detector with a flow speed of 10 mL per minute. This process is continued until the frequency of baseline becomes stable, which indicates the successful deposition of Cuprospinel nanoparticles on the QCM detector. Subsequently, a solution of Cd(II) ions (0.1 µg of Cd(NO_3_)_2_.4H_2_O dissolved in 100 mL double-distilled water, i.e., 3.6 ng/L) flowed above the surface of the Cuprospinel nanoparticles sensor at a flow speed of 10 mL per minute. Figure 1 represented the detection process steps of Cd(II) ions in aqueous solutions using the Cuprospinel nanosensor through the QCM technique.

## 3. Results

### 3.1. Characterization of Cuprospinel Nanoparticles

#### 3.1.1. Identification Analysis

**Figure 2:**
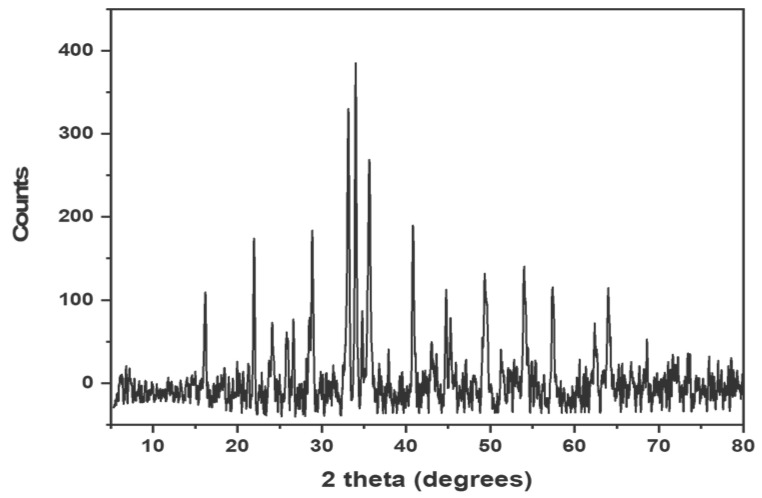
Shows the XRD pattern of synthesized Cuprospinel nanoparticles.

Figure 2 illustrates the fingerprint XRD pattern of synthesized Cuprospinel nanomaterials, which agreed with the results reported by Verwey and Heilmann [58]. As shown in the XRD pattern, the Cuprospinel nanoparticles exhibited characteristic peaks at 30.54, 43.2, 47.3, 53.5, 57.13, 65.9, 75.1, 87.01, and 89.9°. According to the data obtained from XRD analysis, the Cuprospinel nanoparticles were found to have a cubic crystal lattice. It is easily observing the good purity of the synthesized sample, where the XRD pattern lacks any peaks representing the impurities that might be originated from the raw materials.

#### 3.1.2. Structural Analyses

##### Surface Area and Pore Size

**Figure 3:**
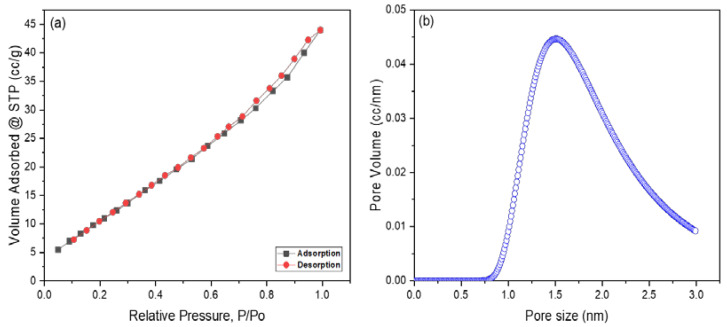
Displays (**a**) N_2_ adsorption–desorption isotherm curve according to the BET method and (**b**) Pore size/volume as determined by the DA method for Cuprospinel nanoparticles.

Figure 3a,b exhibited the N_2_ adsorption–desorption isotherm curve and pore size distribution of Cuprospinel nanoparticles as determined by the BET and DA methods, respectively. The isotherm curve exhibited a typical IV class according to the IUPAC classification of adsorption isotherms, which demonstrates the mesoporous nature of synthesized Cuprospinel nanoparticles [59]. According to de Boer’s classification of hysteresis loops, the Cuprospinel nanoparticles manifested an H3 hysteresis type in which the pores have a slit shape [60]. The BET surface value was found to be 47.3 m^2^/g, the average pore size was 1.5 nm, and the micropore volume was 0.064 cc/g. However, the silt-shape porous, mesopore nature and high BET surface area of synthesized Cuprospinel nanoparticles provide more available sites for detecting the possible largest amount of Cd(II) ions on their surface.

##### Zeta Potential and Particle Size Distribution

**Figure 4:**
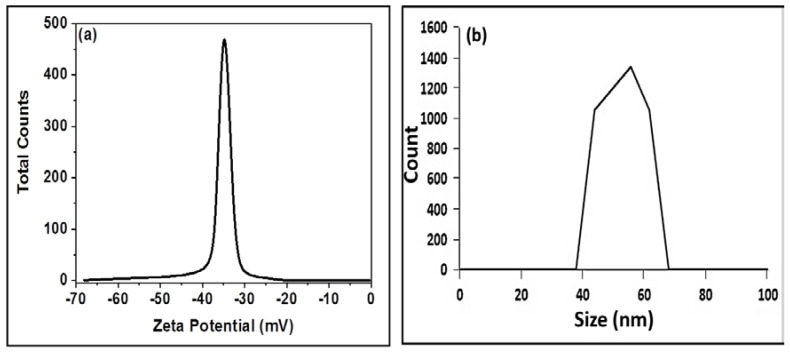
Illustrates (**a**) Charge of Cuprospinel nanoparticles measured by zeta potential and (**b**) particle size distribution of Cuprospinel nanoparticles measured by DLS.

The charge on the surface of synthesized Cuprospinel nanoparticles solid powder in suspension is measured by the zeta potential. A stable, deflocculated, and often the low-viscosity suspension is produced as a result of the high surface charge’s promotion of inter-particle repulsion. Zeta potential also controls how well a particle can interact with other molecules, such as polymeric species and soluble ions. The findings demonstrate that the surface charge of synthesized Cuprospinel nanoparticles is negative at pH neutral and room temperature. The Cuprospinel nanoparticles were found to have a high negative zeta potential value (−36 mV) as was estimated from Figure 4a, indicating that they dispersed well in the neutral pH solutions, which may be due to the inverse spinel structure nature of Cuprospinel particles and increase the electrostatic repulsion between the oxygen ions at the terminal positions of Cuprospinel crystal lattice. Therefore, it could be concluded that the well-dispersed Cuprospinel nanoparticles provide a highly effective surface for detecting the Cd(II) ions. This means the net charge of the Cuprospinel nanoparticles’ surface is negative, leading to strong attraction with Cd cations that carry a positive charge, which results in the good trapping of Cd(II) ions on the surface of the Cuprospinel nanoparticles, i.e., the Cuprospinel nanoparticles offer a highly sensitive and selective nanosensor.

The size distribution profiles of particles in the sub-micron range can be accurately measured using Dynamic Light Scattering (DLS) analysis. This method measures the hydrodynamic diameter of the nanoparticles in solution and offers details on the state of the aggregation of the nanoparticles in solution, making it particularly useful for studying the behavior of nanoparticles in suspensions. Using the DLS approach, the synthesized Cuprospinel nanoparticles were examined, and according to Figure 4b, the synthesized Cuprospinel nanoparticles exhibited an average particle size of about 58 nm. It could be observed that the synthesized Cuprospinel nanoparticles showed a single particle size distribution where no aggregates were formed.

#### 3.1.3. Microscopic Analyses

##### Atomic Force Microscopy (AFM)

**Figure 5:**
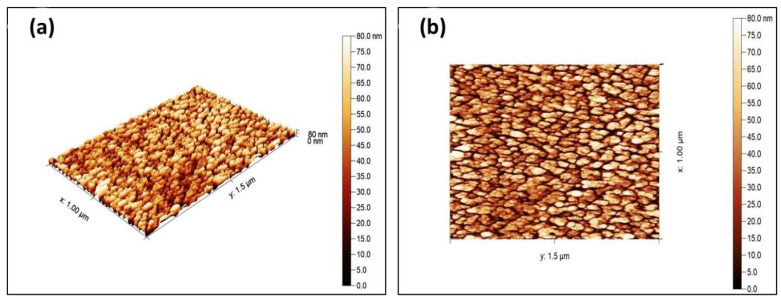
Depicts (**a**) 3D AFM image and (**b**) 2D AFM image of synthesized Cuprospinel nanoparticles.

Atomic force microscopy (AFM) measurements were used to reveal the surface topography of the synthesized Cuprospinel nanoparticles. Figure 5a,b displays 3D and 2D AFM images of synthesized Cuprospinel nanoparticles, respectively. As can be observed, the particles are uniform, homogeneous, and sub-spherically shaped. Additionally, the particles have separate monodisperse distributions and the particle sizes of Cuprospinel nanoparticles are roughly 80 nm. The average grain size of the samples shows that each grain is formed by the aggregation of several nanocrystals.

##### Scanning Electron Microscopy (SEM)

**Figure 6:**
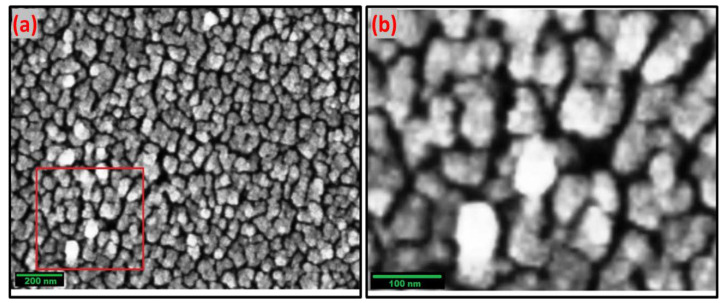
Represents (**a**) 8000× SEM image and (**b**) 160,000× SEM image of Cuprospinel nanoparticles. The selected area by the red square in (**a**) was magnified into (**b**).

Figure 6a,b displays the morphological analysis of the synthesized Cuprospinel nanoparticles at different magnifications. These images demonstrate that the particles had a subspherical shape with distinguishable monodispersity and that their sizes are almost equal, around 80 nm. Moreover, the SEM images also revealed that the samples have a micrometrical aggregation of tiny particles. High-density accumulation may indicate that Cuprospinel nanoparticles have slight pore crystallites on their surface, where the nanoparticles agglomerate and grow into larger assemblies due to their high surface energy. As can be seen, the SEM results support the AFM results, which confirmed the similar particle sizes of the Cuprospinel nanoparticles.

##### Transmission Electron Microscopy (TEM)

**Figure 7:**
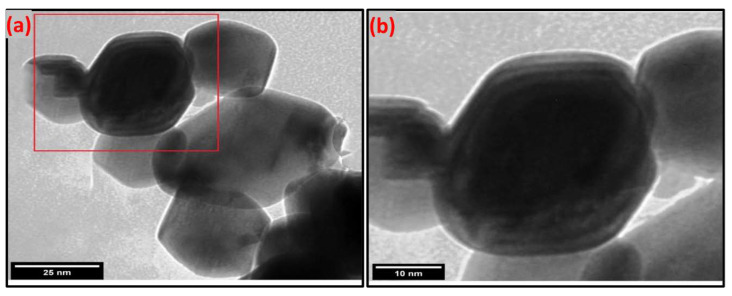
Illustrates (**a**) TEM image of synthesized Cuprospinel nanoparticles and (**b**) zoomed TEM image. The selected area by the red square in (**a**)was zoomed into (**b**).

The particle size, shape, and morphology apparent of the synthesized Cuprospinel nanoparticles were investigated through the transmission electron microscope (TEM). The TEM image (Figure 7a) and its high magnification image (Figure 7b) show that the Cuprospinel nanoparticles have nearly sub-spherical morphology and almost uniform size and distribution. In addition, the Cuprospinel particles seemed non-aggregated and homogeneously dispersed nanoparticles. The average particle size is found to be ranged from 20 to 60 nm approx. Generally, the spinel ferrite particles have a spherical shape with smooth surfaces and narrow size distribution.

### 3.2. Detection of Cd(II) Ion by Cuprospinel Nanoparticles-Based QSM Nanosensor

A Quartz Crystal Microbalance (QCM) (also known as quartz crystal nanobalance (QCN)) measures a mass variation per unit area by measuring the change in the frequency of a quartz crystal resonator. The resonance is disturbed by the addition or removal of a small mass due to oxide growth/decay or film deposition at the surface of the acoustic resonator. In liquid, it is highly effective at determining the affinity of molecules (proteins, in particular) to surfaces functionalized with recognition sites. QCM has also been used to investigate interactions between biomolecules. Frequency measurements are easily formed to high precision; hence, it is easy to measure mass densities down to a level of below 1 μg/cm^2^. The QCM is an extremely sensitive mass balance that measures nanogram to microgram level changes in mass per unit area. The heart of the technology is a quartz disc. Quartz is a piezoelectric material that can be manipulated to oscillate at a defined frequency by applying an appropriate voltage usually via metal electrodes. The frequency of oscillation can be affected by the addition or removal of small amounts of mass onto the electrode surface. This change in frequency can be monitored in real-time to obtain useful information about molecular interactions or reactions taking place at the electrode surface, such as film growth, oxidation, and corrosion/decay.

Sauerbrey demonstrated that the frequency change (Δ*f*) of oscillating quartz could be linearly related to its mass change (adsorbed) (Δ*m*) as expressed by the following relationship [61]:

(1) Δm=−C∗1n∗Δf

where *n* is the overtone number (working area of the crystal), and *C* is a constant that depends on the property of the crystal used (thickness and properties of the quartz crystal). This means that the addition of mass in the nanoscale to the quartz crystal causes a frequency change. Therefore, the measurement of nanogram-scale masses can be achieved. Where any small mass added to the crystal can be treated as an equivalent change in the mass of the quartz crystal itself.

**Figure 8:**
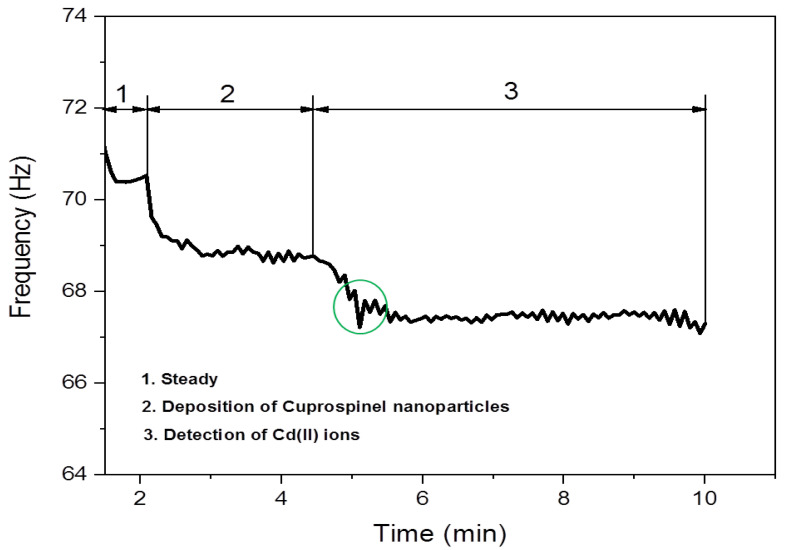
Zone (1) illustrates the stable baseline frequency for an empty QCM detector. Zone (2) represents the successful deposition of Cuprospinel nanoparticles on the surface of the QCM detector. Zone (3) illustrates the successful attaching of Cd(II) ions on the surface of Cuprospinel nanoparticles based-QCM sensorgram. The green circle indicates the suddenly decreasing in the frequency of the QCM sensorgram demonstrating the beginning of Cd(II) detection on the surface of Cuprospinel nanoparticles. This process revealed the ability of Cuprospinel nanoparticles based-QCM as an effective nanosensor for the detection of Cd(II) ion in real-time less than 5 min.

Figure 8 shows schematic diagrams for the full process steps of the deposition of Cuprospinel nanoparticles as a nanosensor material followed by the capturing of Cd(II) ions on its surface along with the curves of the real-time frequency response shifting as a function of measured mass and time. Zone 1 depicts the quartz crystal oscillating at a constant frequency when an appropriate voltage is applied and represents the steady baseline for the clear detector without any additions. Then, the oscillation frequency starts to decrease as the Cuprospinel nanoparticles begin to deposit on the crystal surface. It could observe a sudden decrease in the baseline to low frequency in a constant regime, with time indicating the successful precipitation of sensor material (Cuprospinel nanoparticles) on the surface of quartz crystal. In zone 2, it is easily seen that the presented data exhibited similar behavior to that in zone 1, in which the frequency further decreases to lower values when more Cd(II) ions are deposited on the surface of the nanosensor. As Cd(II) ions are captured at the surface of the sensor, more layers are deposited on the surface of the Cuprospinel nanoparticles, which results in an extension of the thickness of the underlying quartz and leads to a change in the piezoelectrically active crystal area (area between electrodes, cm^2^). Moreover, the baseline appears more oscillated in a response to the correlations of mass to frequency. On the other hand, the response line seems quite smooth for the clear QCM detector (resonant frequency), and then slightly fluctuates with the deposition of Cuprospinel nanoparticles and becomes more fluctuated during the Cd(II) ions detection. These results mean that the added Cd(II) masses are rigidly trapped on the sensor surface and these masses are evenly distributed over the active area of the crystal. Generally, all steps exhibited a constant frequency in an indication of the high stability and sensitivity of a fabricated Cuprospinel nanoparticles-based QCM nanosensor as shown in zone 3, which reveals the stability of frequency over a time reaching 5 min during the detection of Cd(II) ions.

### 3.3. Comparison of the Sensitivity of a Cuprospinel Nanoparticles-Based QSM Nanosensor with Currently Available Methods for the Detection of Cd(II) Ion in the Aqueous Solutions

Table 1 compares the various sensor techniques that have been applied recently to measure Cd(II) ions in aqueous solutions. As can be seen in the table, the Cuprospinel nanoparticles-based QCM nanosensor showed an extremely low LOD value compared to other methods previously reported. This study shows that the Cuprospinel nanoparticle-based QCM nanosensor is an efficient Cd(II) ion detector. Additionally, the use of Cuprospinel nanoparticles offered some advantages to the applied sensing technology based on their significant characteristics such as a large surface area, a high number of recognition sites, and an inverse spinel structure.

In this study, a sensitive sensor-based measuring method that uses QCM technology to selectively and sensitively detect Cd(II) ions at low concentrations is presented. The World Health Organization has established a maximum of 3 μg/L for the amount of Cd(II) ions that are permitted to be present in industrial effluent and drinking water. As a result, a quick, accurate, and reliable approach is needed to measure Cd(II) ions. Because of their special capacity for the real-time monitoring of molecular binding events, QCM sensors are the most often employed chemical sensors. We have proposed a sensitive experimental platform capable of achieving this detection in 10 min. In this work, the advantages of Cuprospinel nanoparticles were leveraged to build the sensor surface for the sensitive detection of Cd(II) ions. These advantages included their environmentally benign synthesis, low cost of fabrication, capacity to scale up, and stability under varied circumstances. The combination of the signal-enhancing properties of nanoparticles and the QCM technique provided sensitive and selective detection with a comparatively low limit of detection value to the sensor system supported the high accuracy of the Cuprospinel nanoparticles-based QCM nanosensor in the detection of the very low concentrations of Cd(II) ions. The limit of detection for the Cuprospinel nanoparticles-based QCM nanosensor has been defined as 3.6 ng/L for the sensitive detection of Cd(II) ions, which is less than the value that was determined by the World Health Organization (WHO).

## 4. Conclusions

In this work, a novel green synthesis of Cuprospinel nanoparticles using *Psidium guajava* L. leaf extract as a reducing agent has been achieved. The synthesized nanoparticles were subjected to different characterization tools such as XRD, N_2_ adsorption–desorption, zeta potential, DLS, AFM, SEM, and TEM. The characterization results revealed that the produced Cuprospinel nanoparticles formed in sub-spherical shapes with particle sizes ranging from 20 to 80 nm. Moreover, the BET surface area and pore size analyses revealed that the synthesized Cuprospinel nanoparticles possess a surface area of 47.3 m^2^/g, an average pore size of 1.5 nm, and a micropore volume of 0.064 cc/g, whereas, the surface of the produced nanoparticles were found to carry a high negative charge with a zeta potential value of −36 mV. In addition, DLS results confirmed the size of particles obtained by the AFM, SEM, and TEM investigations. Subsequently, the synthesized Cuprospinel nanoparticles were examined as a nanosensor material through the QCM technique for the effective detection of Cd(II) ions in aqueous solutions. Accordingly, the novel nanosensor of Cuprospinel nanoparticles-based QCM has proved their high sensitivity and selectivity towards sensing the very low concentrations of Cd(II) ions. The fabricated Cuprospinel nanoparticles-based QCM nanosensor exhibited the ability to detect the Cd(II) ions in concentrations of 3.6 ng/L with high stability continued for 10 min.

## Figures and Tables

**Table 1:** Sensitivity of the Cuprospinel nanoparticles-based QCM nanosensor and sensors-based systems from the literature towards detection of the Cd(II) ion.

No.	Method	LOD Value	Reference
1	Whole-cell biosensors	3 nM	[62]
2	Dispersive liquid-liquid microextraction approach (DLLME) combined with total reflection X-ray spectrometry (TXRF)	0.04 μg/L	[63]
3	Fluorescent probe of CdSe/CdS core–shell quantum dots (QDs)	6 nmol/L	[64]
4	Electrochemical sensor based on Fe_3_O_4_/Bi_2_O_3_/C_3_N_4_ nanocomposites	3 × 10^−9^ mol/L	[65]
5	Electrochemical sensor using a Fe_3_O_4_/G composite	0.08 μg/L	[66]
6	Ion-imprinted surface plasmon resonance sensor	0.01 µg/L	[67]
7	Green synthesized hybrid PVA-chitosan nanocomposite sensor probe	800 ppt	[68]
8	Atomic absorption spectrometry after cloud point extraction	0.44 ng/mL	[69]
9	In situ MID-FTIR-PLS analysis using a polymer inclusion membrane-based sensor	0.45 × 10^−4^ mol/dm^3^	[70]
10	Atomic absorption spectroscopy (AAS) after preconcentration by the XAD-4/GBHD system	0.06–0.50 µg/L	[71]
11	Laser-induced breakdown spectroscopy coupled with chelating resin enrichment	3.6 µg/L	[72]
12	Green solvent-based ultrasonic-assisted dispersive liquid–liquid microextraction and graphite furnace atomic absorption spectrometry	0.2 ng/L	[73]
13	Mercury film silver-based electrode (Hg(Ag)FE) and anodic stripping voltammetric analysis	1.3 × 10^−8^ mol/L	[74]
14	Voltammetric sensing using a bimetallic Hg–Bi supported on poly(1,2 diaminoanthraquinone)/glassy carbon-modified electrode	0.107 μg/L	[75]
15	SnO_2_, MoS_2_, SnO_2_/MoS_2_, SnO_2_–MoS_2_ sensing membrane combination with a fiber-optic Mach–Zehnder interferometer	0–100 µM	[76]
16	Gold-coated reflection-type fiber optic-surface plasmon resonance (Au-coated FO-SPR) sensor		[77]
	BSA/Au	7.1 nM	
	PANI/Au	8.8 nM	
	Chitosan/Au	9.4 nM	
17	Cuprospinel nanoparticles-based QCM nanosensor	3.6 ng/L	The current work

## Data Availability

Not applicable.

## References

[1] Germaninezhad F., Hosseinzadeh R., Tajbakhsh M., Beitollahi A. (2020). Copper ferrite nanoparticles: An effective and recoverable nanomagnetic catalyst for the synthesis of N,N′,N″-trisubstituted guanidines from the addition reaction of anilines to carbodiimide. Micro Nano Lett..

[2] Tran C.V., Quang D.V., Nguyen Thi H.P., Truong T.N., La D.D. (2020). Effective removal of Pb(II) from aqueous media by a new design of Cu–Mg binary ferrite. Acs Omega.

[3] El-Wakeel S.T., Abdel-Karim A., Ismail S.H., Mohamed G.G. (2022). Development of Ag-dendrites@Cu nanostructure for removal of selenium (IV) from aqueous solution. Water Environ. Res..

[4] Galvão W.S., Neto D., Freire R.M., Fechine P.B. (2016). Super-paramagnetic nanoparticles with spinel structure: A review of synthesis and biomedical applications. Solid State Phenomena.

[5] Soufi A., Hajjaoui H., Elmoubarki R., Abdennouri M., Qourzal S., Barka N. (2021). Spinel ferrites nanoparticles: Synthesis methods and application in heterogeneous Fenton oxidation of organic pollutants–a review. Appl. Surf. Sci. Adv..

[6] Wang Y., Miao Y., Li G., Su M., Chen X., Zhang H., Zhang Y., Jiao W., He Y., Yi J. (2020). Engineering ferrite nanoparticles with enhanced magnetic response for advanced biomedical applications. Mater. Today Adv..

[7] Masunga N., Mmelesi O.K., Kefeni K.K., Mamba B.B. (2019). Recent advances in copper ferrite nanoparticles and nanocomposites synthesis, magnetic properties and application in water treatment. J. Environ. Chem. Eng..

[8] Henderson C., Charnock J., Plant D. (2007). Cation occupancies in Mg, Co, Ni, Zn, Al ferrite spinels: A multi-element EXAFS study. J. Phys. Condens. Matter.

[9] Prasad S., Deepty M., Ramesh P., Prasad G., Srinivasarao K., Srinivas C., Babu K.V., Kumar E.R., Mohan N.K., Sastry D. (2018). Synthesis of MFe_2_O_4_ (M = Mg^2+^, Zn^2+^, Mn^2+^) spinel ferrites and their structural, elastic and electron magnetic resonance properties. Ceram. Int..

[10] Tatarchuk T., Bououdina M., Judith Vijaya J., John Kennedy L. (2016). Spinel ferrite nanoparticles: Synthesis, crystal structure, properties, and perspective applications. International Conference on Nanotechnology and Nanomaterials.

[11] Anandan S., Selvamani T., Prasad G.G., Asiri A., Wu J. (2017). Magnetic and catalytic properties of inverse spinel CuFe_2_O_4_. nanoparticles. J. Magn. Magn. Mater..

[12] Zhao J., Xiao P., Han S., Zulhumar M., Wu D. (2022). Preparation of magnetic copper ferrite nanoparticle as peroxymonosulfate activating catalyst for effective degradation of levofloxacin. Water Sci. Technol..

[13] Zuo X., Yang A., Vittoria C., Harris V.G. (2006). Computational study of copper ferrite (CuFe_2_O_4_). J. Appl. Phys..

[14] Dippong T., Levei E.A., Cadar O. (2021). Recent advances in synthesis and applications of MFe_2_O_4_ (M = Co, Cu, Mn, Ni, Zn) nanoparticles. Nanomaterials.

[15] Bhargava G.K., Bhardwaj S., Singh M., Batoo K.M. (2021). Ferrites and Multiferroics: Fundamentals to Applications.

[16] Khan I., Saeed K., Khan I. (2017). Nanoparticles: Properties, applications and toxicities. Arab. J. Chem..

[17] Benelli G. (2019). Green synthesis of nanomaterials. Nanomaterials.

[18] Pandit C., Roy A., Ghotekar S., Khusro A., Islam M.N., Emran T.B., Lam S.E., Khandaker M.U., Bradley D.A. (2022). Biological agents for synthesis of nanoparticles and their applications. J. King Saud Univ.-Sci..

[19] Kuppusamy P., Yusoff M.M., Maniam G.P., Govindan N. (2016). Biosynthesis of metallic nanoparticles using plant derivatives and their new avenues in pharmacological applications–An updated report. Saudi Pharm. J..

[20] Siddiqi K.S., Husen A., Rao R.A. (2018). A review on biosynthesis of silver nanoparticles and their biocidal properties. J. Nanobiotechnol..

[21] Briffa J., Sinagra E., Blundell R. (2020). Heavy metal pollution in the environment and their toxicological effects on humans. Heliyon.

[22] Hamdy A., Mostafa M.K., Nasr M. (2019). Techno-economic estimation of electroplating wastewater treatment using zero-valent iron nanoparticles: Batch optimization, continuous feed, and scaling up studies. Environ. Sci. Pollut. Res..

[23] Hamdy A., Mostafa M.K., Nasr M. (2018). Zero-valent iron nanoparticles for methylene blue removal from aqueous solutions and textile wastewater treatment, with cost estimation. Water Sci. Technol..

[24] Jaishankar M., Tseten T., Anbalagan N., Mathew B.B., Beeregowda K.N. (2014). Toxicity, mechanism and health effects of some heavy metals. Interdiscip. Toxicol..

[25] Tchounwou P.B., Yedjou C.G., Patlolla A.K., Sutton D.J. (2012). Heavy metal toxicity and the environment. Mol. Clin. Environ. Toxicol..

[26] Sadek A.H., Asker M.S., Abdelhamid S.A. (2021). Bacteriostatic impact of nanoscale zero-valent iron against pathogenic bacteria in the municipal wastewater. Biologia.

[27] Mitra S., Chakraborty A.J., Tareq A.M., Emran T.B., Nainu F., Khusro A., Idris A.M., Khandaker M.U., Osman H., Alhumaydhi F.A. (2022). Impact of heavy metals on the environment and human health: Novel therapeutic insights to counter the toxicity. J. King Saud Univ.-Sci..

[28] Hamdy A., Ismail S.H., Ebnalwaled A., Mohamed G.G. (2021). Characterization of superparamagnetic/monodisperse PEG-coated magnetite nanoparticles Sonochemically prepared from the hematite ore for Cd(II) removal from aqueous solutions. J. Inorg. Organomet. Polym. Mater..

[29] Pinot F., Kreps S.E., Bachelet M., Hainaut P., Bakonyi M., Polla B.S. (2000). Cadmium in the environment: Sources, mechanisms of biotoxicity, and biomarkers. Rev. Environ. Health.

[30] Gautam P.K., Gautam R.K., Banerjee S., Chattopadhyaya M., Pandey J. (2016). Heavy metals in the environment: Fate, transport, toxicity and remediation technologies. Nova Sci. Publ..

[31] Engwa G.A., Ferdinand P.U., Nwalo F.N., Unachukwu M.N. (2019). Mechanism and health effects of heavy metal toxicity in humans. Poisoning Mod. World-New Tricks Old Dog.

[32] Rahimzadeh M.R., Rahimzadeh M.R., Kazemi S., Moghadamnia A.-a. (2017). Cadmium toxicity and treatment: An update. Casp. J. Intern. Med..

[33] Ehrampoush M.H., Miria M., Salmani M.H., Mahvi A.H. (2015). Cadmium removal from aqueous solution by green synthesis iron oxide nanoparticles with tangerine peel extract. J. Environ. Health Sci. Eng..

[34] Goyer R.A., Clarkson T.W. (1996). Toxic effects of metals. Casarett Doull’s Toxicol. Basic Sci. Poisons.

[35] Godt J., Scheidig F., Grosse-Siestrup C., Esche V., Brandenburg P., Reich A., Groneberg D.A. (2006). The toxicity of cadmium and resulting hazards for human health. J. Occup. Med. Toxicol..

[36] Balali-Mood M., Naseri K., Tahergorabi Z., Khazdair M.R., Sadeghi M. (2021). Toxic mechanisms of five heavy metals: Mercury, lead, chromium, cadmium, and arsenic. Front. Pharmacol..

[37] Cotruvo J.A. (2017). 2017 WHO guidelines for drinking water quality: First addendum to the fourth edition. J. Am. Water Work Assoc..

[38] Barakat M. (2011). New trends in removing heavy metals from industrial wastewater. Arab. J. Chem..

[39] Yang J., Hou B., Wang J., Tian B., Bi J., Wang N., Li X., Huang X. (2019). Nanomaterials for the removal of heavy metals from wastewater. Nanomaterials.

[40] Pohl A. (2020). Removal of heavy metal ions from water and wastewaters by sulfur-containing precipitation agents. Water Air Soil Pollut..

[41] Vidu R., Matei E., Predescu A.M., Alhalaili B., Pantilimon C., Tarcea C., Predescu C. (2020). Removal of heavy metals from wastewaters: A challenge from current treatment methods to nanotechnology applications. Toxics.

[42] Pugazhenthiran N., Anandan S., Ashokkumar M. (2016). Removal of heavy metal from wastewater. Handb. Ultrason. Sonochem..

[43] Yang L., Hu W., Chang Z., Liu T., Fang D., Shao P., Shi H., Luo X. (2021). Electrochemical recovery and high value-added reutilization of heavy metal ions from wastewater: Recent advances and future trends. Environ. Int..

[44] Türkmen D., Bakhshpour M., Akgönüllü S., Aşır S., Denizli A. (2022). Heavy Metal Ions Removal From Wastewater Using Cryogels: A Review. Front. Sustain..

[45] Hussain A., Madan S., Madan R. (2021). Removal of heavy metals from wastewater by adsorption. Heavy Metals—Their Environmental Impacts and Mitigation.

[46] Qdais H.A., Moussa H. (2004). Removal of heavy metals from wastewater by membrane processes: A comparative study. Desalination.

[47] Nallakukkala S., Rehman A.u., Zaini D.B., Lal B. (2022). Gas Hydrate-Based Heavy Metal Ion Removal from Industrial Wastewater: A Review. Water.

[48] Huang Y., Wu D., Wang X., Huang W., Lawless D., Feng X. (2016). Removal of heavy metals from water using polyvinylamine by polymer-enhanced ultrafiltration and flocculation. Sep. Purif. Technol..

[49] Kanamarlapudi S., Chintalpudi V.K., Muddada S. (2018). Application of biosorption for removal of heavy metals from wastewater. Biosorption.

[50] Hamdy A. (2021). Experimental study of the relationship between dissolved iron, turbidity, and removal of Cu(II) ion from aqueous solutions using zero-valent iron nanoparticles. Arab. J. Sci. Eng..

[51] Abdelmigeed M.O., Sadek A.H., Ahmed T.S. (2022). Novel easily separable core–shell Fe_3_O_4_/PVP/ZIF-8 nanostructure adsorbent: Optimization of phosphorus removal from Fosfomycin pharmaceutical wastewater. RSC Adv..

[52] Katowah D.F., Alsulami Q.A., Alam M., Ismail S.H., Asiri A.M., Mohamed G.G., Rahman M.M., Hussein M.A. (2020). The Performance of Various SWCNT Loading into CuO–PMMA Nanocomposites Towards the Detection of Mn^2+^ Ions. J. Inorg. Organomet. Polym. Mater..

[53] Ismail S.H., Hamdy A., Ismail T.A., Mahboub H.H., Mahmoud W.H., Daoush W.M. (2021). Synthesis and characterization of antibacterial carbopol/ZnO hybrid nanoparticles gel. Crystals.

[54] Lu C., Czanderna A.W. (1984). Applications of Piezoelectric Quartz Crystal Microbalances.

[55] Capparelli R., De Chiara F., Nocerino N., Montella R.C., Iannaccone M., Fulgione A., Romanelli A., Avitabile C., Blaiotta G., Capuano F. (2012). New perspectives for natural antimicrobial peptides: Application as antinflammatory drugs in a murine model. BMC Immunol..

[56] Al-Qasmi N. (2021). Facial eco-friendly synthesis of copper oxide nanoparticles using chia seeds extract and evaluation of its electrochemical activity. Processes.

[57] Saade A.J., Indrajit P., Maria J.C.O., Kadda H., Rosario M., Mohammad J., Abduladheem T., Mahin N., Reza A. (2022). Green synthesis of spinel copper ferrite (CuFe_2_O_4_) nanoparticles and their toxicity. Nanotechnol. Rev..

[58] Verwey E.J.W., Heilmann E.L. (1947). Physical properties and cation arrangement of oxides with spinel structures Locality: Synthetic. J. Chem. Phys..

[59] ALOthman Z.A. (2012). A Review: Fundamental Aspects of Silicate Mesoporous Materials. Materials.

[60] Broekhoff J.C.P. (1979). Mesopore determination from nitrogen sorption isotherms: Fundamentals, scope, limitations. Stud. Surf. Sci. Catal..

[61] Palchoudhury S., Baalousha M., Lead J.R. (2015). Methods for measuring concentration (mass, surface area and number) of nanomaterials. Frontiers of Nanoscience.

[62] He M.-Y., Lin Y.-J., Kao Y.-L., Kuo P., Grauffel C., Lim C., Cheng Y.-S., Chou H.-H.D. (2021). Sensitive and specific cadmium biosensor developed by reconfiguring metal transport and leveraging natural gene repositories. ACS Sens..

[63] Marguí E., Queralt I., Hidalgo M. (2013). Determination of cadmium at ultratrace levels in environmental water samples by means of total reflection X-ray spectrometry after dispersive liquid–liquid microextraction. J. Anal. At. Spectrom..

[64] Wang D., Gao F., Wang X., Ning X., Wang K., Wang X., Wei Y., Fujita T. (2022). Detection of Cd^2+^ in Aqueous Solution by the Fluorescent Probe of CdSe/CdS QDs Based on OFF–ON Mode. Toxics.

[65] Pu Y., Wu Y., Yu Z., Lu L., Wang X. (2021). Simultaneous determination of Cd^2+^ and Pb^2+^ by an electrochemical sensor based on Fe_3_O_4_/Bi_2_O_3_/C_3_N_4_ nanocomposites. Talanta Open.

[66] Lee S., Oh J., Kim D., Piao Y. (2016). A sensitive electrochemical sensor using an iron oxide/graphene composite for the simultaneous detection of heavy metal ions. Talanta.

[67] Bakhshpour M., Denizli A. (2020). Highly sensitive detection of Cd(II) ions using ion-imprinted surface plasmon resonance sensors. Microchem. J..

[68] Boruah B.S., Biswas R., Baishya U. (2020). Ultrasensitive trace determination of cadmium through a green synthesized hybrid PVA-Chitosan nanocomposite. Plasmonics.

[69] Golbedaghi R., Jafari S., Yaftian M.R., Azadbakht R., Salehzadeh S., Jaleh B. (2012). Determination of cadmium (II) ion by atomic absorption spectrometry after cloud point extraction. J. Iran. Chem. Soc..

[70] González-Albarrán R., de Gyves J., Rodríguez de San Miguel E. (2020). Determination of Cadmium (II) in Aqueous Solutions by In Situ MID-FTIR-PLS Analysis Using a Polymer Inclusion Membrane-Based Sensor: First Considerations. Molecules.

[71] Ghorbani A., Khosropour Z., Najarian S. Determination of cadmium and lead ions in water sample by AAS after preconcentration by XAD-4/GBHD system. Proceedings of the 15th International Conference on Heavy Metals in the Environment.

[72] Tian H., Jiao L., Dong D. (2019). Rapid determination of trace cadmium in drinking water using laser-induced breakdown spectroscopy coupled with chelating resin enrichment. Sci. Rep..

[73] Ghorbani M., Akbarzade S., Aghamohammadhasan M., Seyedin O., Lahoori N.A. (2018). Pre-concentration and determination of cadmium and lead ions in real water, soil and food samples using a simple and sensitive green solvent-based ultrasonic assisted dispersive liquid–liquid microextraction and graphite furnace atomic absorption spectrometry. Anal. Methods.

[74] Adamczyk M., Grabarczyk M. (2019). Simple, insensitive to environmental matrix interferences method of trace cadmium determination in natural water samples. Ionics.

[75] Hassan S. (2020). Single and simultaneous voltammetric sensing of lead(II), cadmium(II) and zinc(II) using a bimetallic Hg-Bi supported on poly (1,2-diaminoanthraquinone)/glassy carbon modified electrode. Sens. Bio-Sens. Res..

[76] Chen H., Yang X., Feng W. (2021). Cadmium-ion detection: A comparative study for a SnO_2_, MoS_2_, SnO_2_/MoS_2_, SnO_2_-MoS_2_ sensing membrane combination with a fiber-optic Mach–Zehnder interferometer. Appl. Opt..

[77] Şolomonea B.-G., Jinga L.-I., Antohe V.-A., Socol G., Antohe I. (2022). Cadmium Ions’ Trace-Level Detection Using a Portable Fiber Optic—Surface Plasmon Resonance Sensor. Biosensors.

